# Screen time and adolescents' mental health before and after the COVID-19 lockdown in Switzerland: A natural experiment

**DOI:** 10.3389/fpsyt.2022.981881

**Published:** 2022-11-16

**Authors:** Laura Marciano, Kasisomayajula Viswanath, Rosalba Morese, Anne-Linda Camerini

**Affiliations:** ^1^Harvard T. H. Chan School of Public Health and Dana-Farber Cancer Institute, Boston, MA, United States; ^2^Faculty of Biomedical Sciences, Università della Svizzera italiana, Lugano, Switzerland; ^3^Faculty of Communication, Culture and Society, Università della Svizzera italiana, Lugano, Switzerland; ^4^Institute of Public Health, Università della Svizzera italiana, Lugano, Switzerland

**Keywords:** screen time, social media, mental health, COVID-19, lockdown, longitudinal, natural experiment, adolescents

## Abstract

**Background:**

During the COVID-19 lockdown in 2020, adolescents' mental health was largely undermined. A general increment in screen time was reported. However, the long-term effects of the latter on adolescents' mental health are still little explored.

**Methods:**

In the present natural experiment, we investigated these effects using longitudinal data collected before and after the first lockdown in Switzerland. Data come from 674 Swiss adolescents (56.7% females, M_age_ = 14.45, SD_age_ = 0.50) during Spring 2019 (T1) and Autumn 2020 (T2) as part of the longitudinal MEDIATICINO study. Self-reported mental health measures included somatic symptoms, inattention, anxiety, irritability, anger, sleep problems, obsessive-compulsive symptoms, loneliness, and depression. Measures for screen-media activities included time spent on the Internet, smartphones, social media, video gaming, instant messaging, and television viewing. They were all assessed at T1 and T2.

**Results:**

Paired-sample *t*-tests with Bonferroni's correction showed that most mental health problems increased over time with an overall medium effect size (Hedge's *g* = 0.337). In particular, medium effect sizes were found for anxiety, depression, and inattention; small-to-medium effect sizes were reported for loneliness, sleep problems, and obsessive-compulsive symptoms; and a small effect size was found for somatic symptoms. Screen-media activities increased, with the exception of television viewing and video gaming. The results of the hierarchical regression analyses showed that, controlling for covariates, increased time spent on social media – calculated as the difference between T2 and T1 – was the only screen-media activity significantly associated with worse mental health at T2 (β = 0.112, *p* = 0.016). More time spent in structured media activities like television viewing diminished levels of inattention (β = −0.091, *p* = 0.021) and anxiety (β = −0.093, *p* = 0.014). Among covariates, being female, experiencing two or more life events, having mental health problems at T1, and using screens for homeschooling negatively influenced mental health at T2.

**Conclusion:**

These results align with literature indicating a small but negative effect of social media time on mental health. Underlying mechanisms are manifold, including increased exposure to COVID-19 news, heightened fear of missing out, social comparison, and time-displaced for activities such as physical activity and green time. However, in line with the *structured days hypothesis*, getting involved in media-structured activities like television viewing might protect against mental health symptoms.

## Introduction

On March 11, 2020, the WHO declared COVID-19 a worldwide pandemic (1). Within a few weeks, countries around the globe, including Switzerland, adopted a nationwide lockdown as one of the most drastic containment measures to counter the spread of the disease. For adolescents, the measures included school closure, the interruption of all extracurricular activities such as sports and music lessons, and strict social distancing from contacts outside their own households. By June 2020, the lockdown in Switzerland was lifted, and schools moved to hybrid formats of in-person and online lectures. Yet, in-person extracurricular activities remained forbidden and social distancing measures were highly recommended. The disruption of adolescents' everyday life and social contacts has profoundly impacted their wellbeing and mental health (2–7), especially considering that adolescence is a developmental period with many challenges. The challenges include discovering and understanding of own identity, establishing a sense of independence, forming important close and intimate relations, and achieving important goals or decisions for the future (8). Biologically, brain regions undergo significant changes (9, 10). Adolescence is a period of higher vulnerability and propensity to carry out risk-taking as well as reward-oriented and novelty-seeking behaviors (11), especially if a social component is involved (12). The gradual development of the cognitive-control system helps to augment adolescents' capacity to self-regulate their behaviors, particularly their emotions (13, 14). This stage of life is also the most common time for the onset of psychological difficulties, including externalizing (e.g., conduct disorder, substance use, and abuse) and internalizing (e.g., depression, anxiety) problems. Indeed, half of the mental health disorders with long-lasting effects start in mid-adolescence (15–17), which overlaps with the age of the heaviest digital media use (18).

Numerous systematic reviews and meta-analyses have reported an increase in mental health problems such as depression, stress, anxiety, loneliness, and attention problems during the COVID-19 pandemic in adolescents (3, 6, 7, 19–33). In particular, Catling et al. (21) underlined how stress represented an essential factor that mediates the increase in depression and anxiety (34). Other factors related to lower mental health included, among others, school closures and the associated disruption of daily routines (35), changes in lifestyle behaviors such as sleep, physical activity, nutrition, and substance use (36, 37), lack of social contact, especially with peers (38), conflict with parents and domestic violence (3, 39), and (excessive) screen time (6, 40, 41). The latter is of particular interest since a more nuanced look at the effects of screen time revealed that it was not bad *per se*, but its effects depended on the type of screen-media activities. For example, a systematic review and meta-analysis of 30 studies (41) revealed that social media use mitigated feelings of loneliness and stress during the pandemic when its use included one-to-one or one-to-few communications (e.g. VoIP apps such as Skype, Viber, WhatsApp), and when online disclosure was promoted in the context of reciprocal friendships. In addition, positive feedback and communication, including humor, augmented feelings of social connection and happiness. On the other hand, one-to-many communication in the form of browsing through other users' social media profiles or websites was associated with worse mental health as users experienced, among others, fear of missing out (FoMO), a tendency to ruminate and distress due to being repeatedly exposed to an overload of negative information about the pandemic (41). There was a three-fold increase in mental health symptoms when social media time augmented drastically (e.g., 3 h) with respect to the pre-pandemic period, and symptoms were worse in adolescents already experiencing mental health problems. Additionally, screen time – when not related to social activities – was associated with lower social wellbeing, including less perceived social support and a higher feeling of loneliness (41).

The above-mentioned review has furthermore revealed that many of the included studies, 24 out of 30, used a cross-sectional design, thus limiting conclusions on the actual effect of screen-media use on adolescents' mental health. The six longitudinal studies showed mixed results but also differed regarding the study design, observed time period, and screen time measurement. For example, a day-by-day assessment over 2 weeks during the lockdown identified increased levels of social media use and television viewing while gaming remained relatively stable (42). Furthermore, applying a week-by-week assessment for 3 weeks during the lockdown. Furthermore, Fumagalli et al. (43) found that social networking and VoIP app usage increased during the lockdown. Looking at the different type of screen-media activities, this study with weekly assessments found that social networking app use was positively associated with loneliness in the subsequent week (43). Yet another study on 844 Swiss children and adolescents, screened monthly over 5 months – between Autumn 2020 and Spring 2021 after strict lockdown measures were lifted – found stable levels of electronic device use within children and adolescents (44). Although participants spent an average of 2 h and 40 min of leisure screen time at each time point, adolescents aged 15 or older were the ones spending more time, with an average of 4 h and 20 min. With respect to the development of mental health problems, a study with two assessments, including 1,64,101 Chinese college students at the onset of the pandemic and 68,685 participants about 2.5 months later, showed that acute stress diminished, whereas depressive and anxiety symptoms augmented. To note, social media exposure was a risk factor for mental health problems, especially when participants spent more than 3 h per day on social media platforms (45). Furthermore, the authors found a positive effect of social media use on stress and anxiety symptoms (45). Also Rosen et al. (46) found no effect of screen time and a negative effect of news consumption on internalizing symptoms among adolescents.

The abovementioned longitudinal studies lacked an accurate baseline measure before the pandemic's onset. Of course, nobody could have expected the COVID-19 pandemic, and only a few longitudinal studies that had already started before the pandemic could collect follow-up data, thus becoming “natural experiments” and providing a comprehensive picture of what has changed with the emergence of a life-changing event of this scale. Available natural experiments with screen time data collected in young people stemmed from Germany (47, 48), China (49), and Australia (50). For example, drawing from 1711 4- to 17-year-olds, Schmidt et al. (47) revealed an increase in television viewing, gaming, and recreational Internet use during the lockdown in Germany compared to the pre-pandemic assessment, especially in the adolescent group. However, the study did not include measures of mental health. Another study, using the same panel data from Germany, found an increase in screen time among adolescent girls and boys and a decrease in health-related quality of life, capturing both physical and psychological wellbeing indicators. However, pre-pandemic screen time did not influence the negative changes in health-related quality of life, which was mainly predicted by previous levels on the same variable (48). Xiang et al. (49) surveyed 2426 children and adolescents aged 6 to 17 years between January and March 2020 (immediately before and during the first lockdown in China). The authors reported an increase from 7 to 31% of children and adolescents with long leisure screen hours (defined as more than 2 h per day). However, also in this case, no data on mental health were reported. Finally, Magson et al. (50) collected data on 248 Australian adolescents aged 14 years. They found a significant increase in depression and anxiety and a decrease in life satisfaction from before the pandemic to 2 months into the pandemic with a modest effect size (from 0.2 to 0.6 standard deviations). Media exposure measures included traditional and social media use. In particular, television viewing and newspaper reading about COVID-19 had a negative relationship with changes in anxiety, and no relationship with changes in depression and life satisfaction, whereas social media exposure during the lockdown was unrelated to changes in mental health outcomes.

Overall, there are two main shortcomings of the abovementioned natural experiments: (i) they considered a comparably short time frame of a few weeks into the pandemic, thus lacking evidence on long-term changes in screen-media activities and mental health; (ii) three of the four studies looked at changes in screen time in relation to other variables than mental health (like physical activity) and only one (50) looked at the bidirectional associations between changes in mental health and screen time measures (although the latter was limited to overall traditional and social media exposure). In the present natural experiment, we aimed to investigate the long-term effects of the COVID-19 pandemic, over 1 year, on adolescents' mental health by using longitudinal data collected before and after the lockdown in Switzerland. In particular, we wanted to estimate how adolescents' mental health and screen-media use changed from Spring 2019 to Autumn 2020 and (ii) how changes in screen-media use at T1 influenced adolescents' mental health in Autumn 2020. The study leverages the unforeseen COVID-19 pandemic that emerged during an ongoing longitudinal study with adolescents in Switzerland.

## Methods

### Study design and sample

For the current paper, we drew on data collected in spring 2019 (wave 6) and autumn 2020 (wave 7), following labeled as “T1” and “T2”, of the MEDIATICINO prospective cohort study (www.mediaticino.usi.ch). The study started in the spring of 2014 with a cohort of 9- to 10-year-old students in elementary schools randomly selected across the entire Canton Ticino, Southern Switzerland. In 2020, participants entered high schools, and additional students were randomly invited to compensate for drop-outs. The study sample represents approximately one-third of the entire underlying population of that age in Switzerland. They have been followed up once a year through the middle and high school years. Data for each wave were collected through a paper-and-pencil questionnaire distributed by teachers at school and matched with the help of a unique identifier, which was assigned to each student by the regional education administration. This procedure assured anonymity during data collection and analysis. Ethical approval of the study was received by the regional education administration. For further information on the study design, see also Camerini et al. (51).

Of the distributed questionnaires (*n* = 1391 at T1 and *n* = 1476 at T2), schools returned 1224 at T1 (88%) and 1088 at T2 (74%). The main reasons for sample attrition were students being absent from school during the day of data collection, school dropouts, or moving to another Swiss Canton or country. When matching participants from T1 and T2, *n* = 575 did not participate in both waves or had more than 10% missing data of the included variables. Two participants were removed due to outlier data defined as z-scores > |−3.5| on the continuous variables (52).

### Measures

Measures were translated from English into Italian when necessary, and independent back-translation was performed to assure linguistic validity. A complete list of items and response options can be found in Supplementary Table 1. Means and standard deviations for each concept at each measured wave are summarized in Table 1.

**Table 1:** Means, standard deviations, and paired-samples t-tests results.

**Variables**	**Before COVID-19 lockdown M(SD)_T1_**	**After COVID-19 lockdown M(SD)_T2_**	***t***	***df***	***P*-value**	**Hedges' g**
**Psychopathological symptoms**						
Depression	0.72 (0.71)	1.07 (0.82)	10.189	668	< 0.001	0.394
Loneliness	2.19 (0.79)	2.37 (0.77)	5.374	669	< 0.001	0.207
Inattention	2.24 (0.85)	2.54 (0.93)	7.779	668	< 0.001	0.301
Sleep problems	2.29 (0.98)	2.51 (0.95)	5.528	668	< 0.001	0.214
Anxiety	2.33 (1.07)	2.91 (1.18)	12.099	667	< 0.001	0.468
Somatic problems	2.47 (1.08)	2.57 (1.03)	2.245	667	0.025	0.087
Irritability	2.34 (1.14)	2.41 (1.11)	1.271	661	0.204	0.049
Anger	1.83 (1.02)	1.87 (1.07)	0.687	662	0.492	0.027
Obsessive-compulsive disorder	2.10 (1.15)	2.37 (1.22)	4.668	660	< 0.001	0.181
**Overall mental health problems**	2.05 (0.67)	2.29 (0.68)	8.76	673	< 0.001	0.337
**Screen-media activities**						
Internet use	4.16 (1.85)	4.83 (1.77)	8.442	651	< 0.001	0.330
Smartphone use	4.08 (1.86)	4.71 (1.73)	8.395	659	< 0.001	0.327
Messaging	2.76 (1.89)	2.99 (1.77)	3.118	660	0.002	0.121
Video gaming	1.55 (1.95)	1.36 (1.74)	−2.812	660	0.005	−0.109
Social media use	3.04 (1.95)	3.31 (1.67)	3.560	659	< 0.001	0.139
Television viewing	2.61 (1.77)	1.73 (1.50)	−10.784	640	< 0.001	−0.426
**Overall screen time**	3.03 (1.37)	3.15 (1.12)	2.547	673	0.011	0.098
**Social screen time** (excl. video gaming, television viewing)	3.50 (1.66)	3.95 (1.53)	7.407	674	< 0.001	0.285

#### Mental health

Mental health was measured at T1 and T2 with an adapted version of the Diagnostic and Statistical Manual of Mental Disorders, Fifth Edition (DSM-5) Self-Rated Level 1 Cross-Cutting Symptom Measure for children aged 11–17 yrs. This checklist asks if, in the past month, participants experienced psychopathological symptoms on a scale from 1 to 5, where 1 = never (none), 2 = rarely (slight), 3 = several days (mild), 4 = more than a half of days (moderate), and 5 = almost every day(severe). Symptoms included somatic symptoms (1 item), inattention (3 items), anxiety (2 items), irritability (1 item), anger (1 item), sleep problems (3 items), and obsessive-compulsive disorder (OCD) symptoms (1 item). Multi-item indicators were averaged to obtain one score for each symptom (inattention α_T1 =_ 0.791, α_T2 =_ 0.807; anxiety r_T1 =_ 0.656, r_T2 =_ 0.683; sleep problems α_T1 =_ 0.690, α_T2 =_ 0.618). Higher values indicate higher levels of mental health problems.

#### Loneliness

Loneliness was measured at T1 and T2 with the 3-item version of the UCLA Loneliness Scale recommended for large population-based surveys (53). Items were measured on a scale from 1 = never to 4 = always (α_T1 =_ 0.752, α_T2 =_ 0.729).

#### Depression

Depression was measured at T1 and T2 with seven items from the Center for Epidemiologic Studies Depression Scale [CES-D; (54)]. Items were measured on a scale from 1 = not at all to 4 = a lot (α_T1 =_ 0.911, α_T2 =_ 0.918).

#### Screen-media activities

Screen-media activities were measured at T1 and T2 as time spent in different online activities on “a typical school day” and on “a typical weekend day”. Response options were 0 = never, 1 = up 0.5 h, 2 = 0.5 h−1h, 3 = 1–1.5h, 4 = 1.5–2h, 5 = 2–3h, 6 = 3–4h, 7 = 4–5h, and 8 = 5h or more. Activities included: Internet use, smartphone use, social media use, video gaming, instant messaging, and television viewing. For each activity, we calculated a weighted average of daily use by applying the formula [(screen-media activity on a weekday^*^5 + screen-media activity on a weekend day^*^2)/7)]. In addition, we created an overall screen time measure by calculating an average of the six weighted averages (α_T1 =_ 0.817, α_T2 =_ 0.737).

#### Covariates

Covariates assessed at T1 included gender coded as 0 = male and 1 = female. At T2, we assessed the current subjective socio-economic status (SES) measured with the single item “How would you evaluate the financial situation of your family?” on a scale from 0 = very wealthy to 4 = definitely not wealthy. For further analyses, answers were categorized as 0 = wealthy or very wealthy and 1 = not wealthy or definitely not wealthy. In addition, we assessed the experience of life events. These included parents' divorce, a parent with a new partner, a parent's loss of his/her job, a family member who had deceased or had severe disease, the worsening of a significant relationship, or any other important negative life events. For each of these events, participants reported if it happened to them during the past year, coded as 0 = no and 1 = yes. We categorized all events into 0 = none, 1 = one, and 2 = two or more two life events for the analyses. Also, we controlled for the at-home living situation in 2020 by asking the following question: “Who is living at home with you?”. Answer options, coded as 0 = not present and 1 = present, included mother, father, older sibling(s), younger sibling(s), mother's partner, father's partner, grandparents, and others. We summed up the answers by creating two categories 0 = one or two other people and 1 = three or more other people. Finally, we controlled for the time spent on screens for homeschooling by asking participants, “In general, how often do you use the following device (laptop/smartphone/tablet) for homeschooling?”. Answers options ranged from 1 = never to 5 = always.

### Analytical plan

First, we described the levels of cross-cutting mental health symptoms, loneliness, depression, and screen-media activities before and after the COVID-19 lockdown in Switzerland. Second, after checking that data were normally distributed according to the values of skewness and kurtosis, we estimated how mental health changed over time by running paired-sample t-tests to compare mental health symptoms, loneliness, and depression between T1 and T2. A Bonferroni correction was applied to ensure that possible differences were not due to chance. To better interpret the magnitude of the difference between pre- and post- COVID-19 lockdown assessments, we computed Hedge's *g* as a measure of effect size. The magnitude of the effect was interpreted as small = 0.10, mediu*M* = 0.30, and large = 0.50 effect (55). Third, we computed a difference index of screen-media activities (Δ_T2 − T1_), i.e., subtracting the time spent on screen media of T1 from T2. Thus, positive values in the Δ index indicate increased time spent with screen media from T1 to T2. We computed Δ indices for each screen media activity and overall screen time. Fourth, we computed bivariate Pearson's correlation coefficients for all measures (see Table 2). Fifth and last, we ran hierarchical regression analyses with mental health at T2 as the outcome by adding a new block of predictors at each step, in addition to the baseline model with only covariates (Model 1), including gender, life events, subjective SES, the at-home living situation at T2, and the use of screens for homeschooling at T2. In particular, mental health at T1 was further added as a predictor in Model 2, and the Δ indices in screen-media activities were additional predictors in Model 3. To avoid problems of multicollinearity, a model including covariates, overall mental health problems at T1, and the Δ index for overall screen time was run separately (Model 4; see Table 3).

**Table 2:** Correlation table of all the variables included in the regression models.

	**1**	**2**	**3**	**4**	**5**	**6**	**7**	**8**	**9**	**10**	**11**	**12**	**13**
1. Mental health problems at T1	1												
2. Mental health problems at T2	0.475**	1											
3. Internet use_Δ*T*2−*T*1_	−0.112**	−0.004	1										
4. Smartphone use_Δ*T*2−*T*1_	−0.080*	0.017	0.646**	1									
5. Social media g_Δ*T*2−*T*1_	−0.205**	0.011	0.469**	0.556**	1
6. Messaging_Δ*T*2−*T*1_	−0.142**	0.008	0.395**	0.504**	0.584**	1							
7. Television viewing _Δ*T*2−*T*1_	−0.084*	−0.086*	0.067	0.072	0.164**	0.145**	1						
8. Video gaming_Δ*T*2−*T*1_	0.061	0.046	0.185**	0.164**	0.121**	0.163**	0.119**	1					
9. Overall social screen time _Δ*T*2−*T*1_	−0.169**	0.008	0.788**	0.843**	0.810**	0.770**	0.137**	0.190**	1				
10. At-home living situation at T2	−0.058	−0.012	0.037	0.039	0.042	0.064	0.006	0.047	0.056	1			
11. Subjective SES at T2	0.018	0.014	−0.069	−0.066	−0.012	−0.032	−0.063	0.006	−0.042	−0.076*	1		
12. Life-changing events at T2	0.214**	0.294**	0.016	0.021	−0.033	−0.015	−0.015	0.038	0.004	−0.168**	0.142**	1	
13. Use of screens for home schooling at T2	0.028	0.126**	0.132**	0.061	0.076	0.093*	0.027	0.014	0.117**	−0.057	−0.015	0.043	1
14. Gender	0.277**	0.306**	−0.057	−0.049	−0.090*	−0.045	−0.046	0.232**	−0.071	−0.019	−0.033	0.149**	0.034

^*^p < 0.05,

^**^p < 0.01.

**Table 3:** Hierarchical regression results with Mental health problems at T2 as outcome.

	**Model 1**				**Model 2**				**Model 3**				**Model 4**			
	**B**	**(SE)**	**Beta**	***P*-value**	**B**	**(SE)**	**Beta**	***P*-value**	**B**	**SE**	**Beta**	***P*-value**	**B**	**(SE)**	**Beta**	***P*-value**
(Constant)	1.505	0.120		0.000	0.866	0.128		< 0.001	0.806	0.127		< 0.001	0.865	0.122		< 0.001
Gender (1 = female)	0.359	0.052	0.263	< 0.001	0.246	0.050	0.180	< 0.001	0.264	0.050	0.193	< 0.001	0.226	0.047	0.163	< 0.001
Life events at T2 (*n* = 1)	0.262	0.064	0.168	< 0.001	0.199	0.060	0.127	< 0.001	0.216	0.059	0.138	< 0.001	0.226	0.056	0.145	< 0.001
Life events at T2 (*n* ≥ 2)	0.453	0.063	0.304	< 0.001	0.337	0.059	0.226	< 0.001	0.343	0.059	0.230	< 0.001	0.309	0.056	0.204	< 0.001
Subjective SES at T2 (1 = not wealthy)	0.018	0.054	0.013	0.738	0.016	0.050	0.011	0.751	0.016	0.050	0.011	0.748	−0.018	0.047	−0.013	0.694
At-home living situation at T2 (1 = with 3 or more other people)	0.092	0.056	0.064	0.097	0.102	0.051	0.071	0.046	0.099	0.051	0.069	0.051	0.069	0.049	0.046	0.163
Use of screens for home schooling at T2	0.111	0.035	0.119	0.002	0.105	0.033	0.113	0.001	0.095	0.033	0.102	0.004	0.094	0.031	0.100	0.003
Overall mental health problems at T1					0.372	0.037	0.371	< 0.001	0.390	0.037	0.389	< 0.001	0.401	0.036	0.394	< 0.001
Internet use_Δ*T*2−*T*1_									−0.006	0.016	−0.017	0.715	–	–	–	–
Smartphone use_Δ*T*2−*T*1_									0.007	0.018	0.019	0.699	–	–	–	–
Messaging_Δ*T*2−*T*1_									0.015	0.016	0.041	0.364	–	–	–	–
Video gaming_Δ*T*2−*T*1_									−0.020	0.015	−0.050	0.171	–	–	–	–
Social media use_Δ*T*2−*T*1_									0.041	0.017	0.112	0.016	–	–	–	–
Television viewing_Δ*T*2−*T*1_									−0.023	0.012	−0.069	0.053	–	–	–	–
Overall social screen time_Δ*T*2−*T*1_													0.036	0.015	0.083	0.014
*R* ^2^	0.188**				0.311**				0.326**				0.301**			

^**^p < 0.01. _Δ*T*2−*T*1_ = difference index between T2 and T1 (SE) = standard error.

## Results

The final analytical sample was composed of 674 Swiss adolescents, 56.7% (*n* = 382) females, with a mean age of 14.45 years at T1 (SD = 0.50). At T2, most of them reported being wealthy or very wealthy (*n* = 410, 60.8%). Also, 301 students (44.7%) reported having not experienced any life event, whereas 173 (25.7%) and 190 (28.2%) indicated having experienced one and two or more life events, respectively. In particular, the worsening of a significant relationship was the most reported one (31.8%). Concerning the at-home living situation, 31.5% of participants reported living with one (6.2%) or two (25.2%) other people, and the remaining adolescents with three (59.2%) or more (9.4%). Both the laptop (*M* = 3.45, SD = 1.07) and the smartphone (*M* = 3.49, SD = 1.16) were used quite often for homeschooling, whereas the tablet was used less for this aim (*M* = 1.66, SD = 1.10).

**Figure 1:**
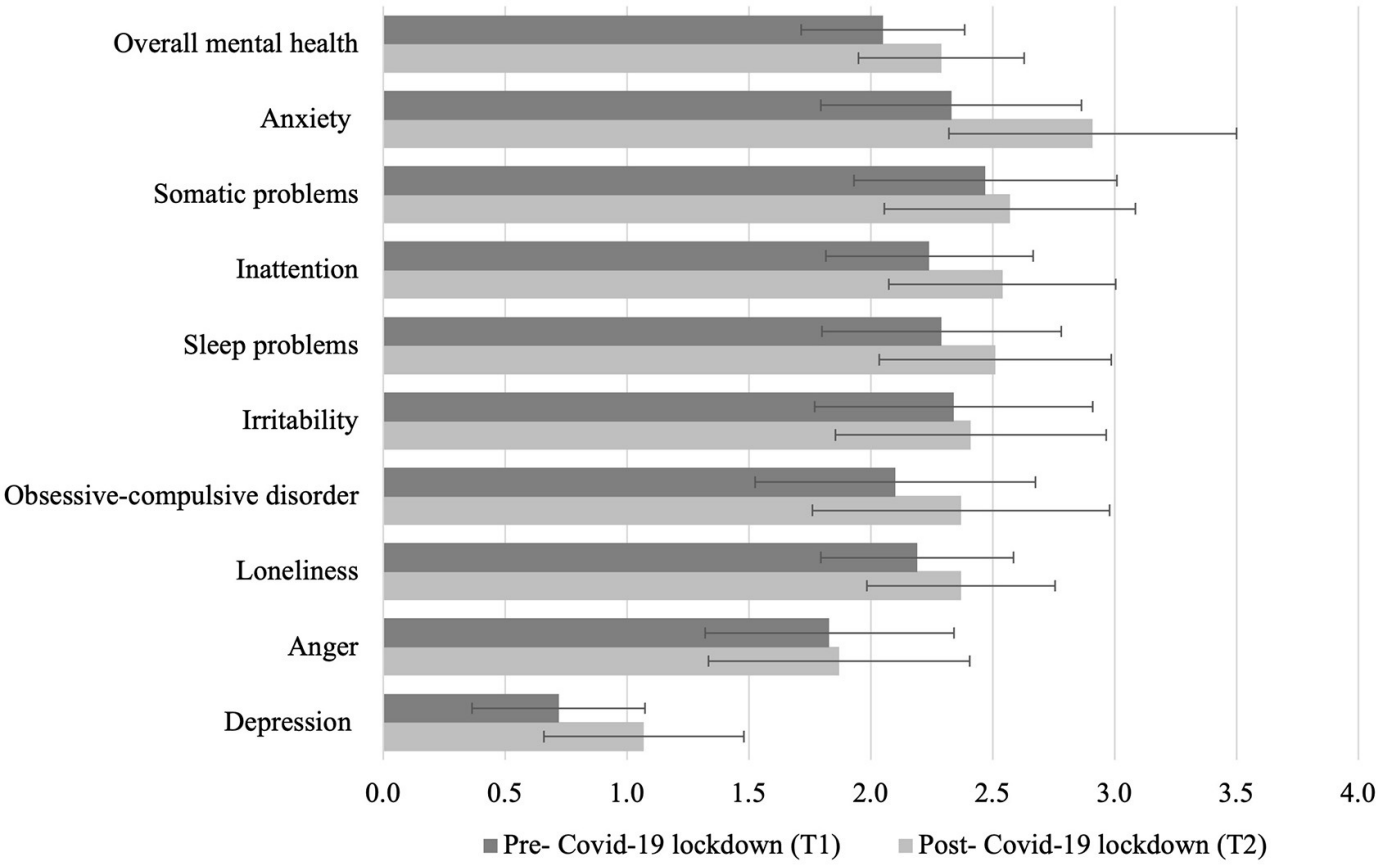
Mental health before and after the COVID-19 lockdown in Switzerland. Means and standard deviations are shown.

The results of the paired-sample *t*-tests showed that most mental health problems increased from T1 to T2 (see Table 1 and Figure 1) with an overall medium effect size (*g* = 0.337). In particular, medium-to-large effect sizes were reported, in descending order, for anxiety (*g* = 0.468), depression (*g* = 0.394), and inattention (*g* = 0.301); small-to-medium effect sizes were found for sleep problems (*g* = 0.214), loneliness (*g* = 0.207), and OCD symptoms (*g* = 0.181); and small effect size for somatic problems (*g* = 0.087), although the latter was not significant when Bonferroni's correction was applied (*p* < 0.005). No differences were found with respect to irritability and anger.

**Figure 2:**
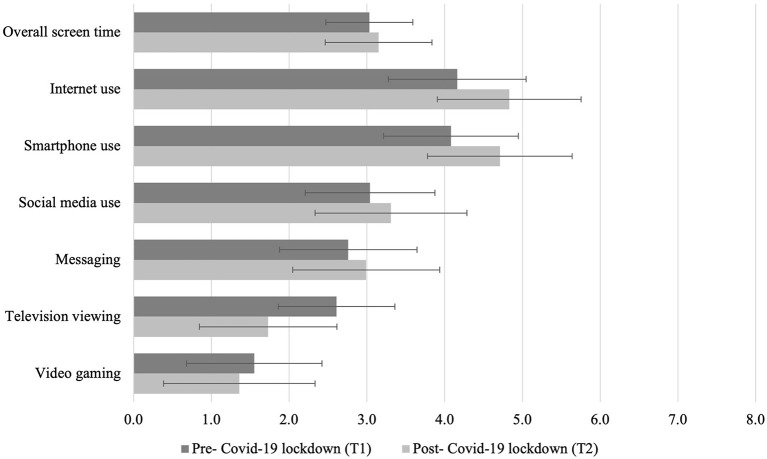
Daily duration of screen-media activities before and after the COVID-19 lockdown in Switzerland. Means and standard deviations are shown.

Pairwise comparisons further showed that screen-media activities increased, except for television viewing and video gaming, which decreased from the pre-lockdown to the post-lockdown assessment (see Table 1 and Figure 2). In particular, a medium effect was found for Internet use (*g* = 0.330) and smartphone use (*g* = 0.327), whereas a small effect was found for social media use (*g* = 0.139) and instant messaging (*g* = 0.121). Television viewing decreased with a medium-to-large effect (*g* = –0.426), whereas videogaming decreased with a small effect (*g* = −0.109) in the same direction. All differences were statistically significant, even after applying Bonferroni's correction (*p* < 0.007). Overall screen time augmented with a small effect size (*g* = 0.098). However, when TV viewing and videogaming (which decreased) were excluded from overall screen time, changes in screen time were of medium size (*g* = 0.285). Thus, we opted to use the latter measure of screen time as more representative of online activities, and we further referred to this overall average of time spent on the Internet, social media, messaging and on the smarpthone as “social” screen time.

The correlation table (see Table 2) shows that mental health problems at T1 were positively related to mental health problems at T2, but negatively related to the difference indices of all screen-media activities with the exception of videogaming. Conversely, mental health problems at T2 positively correlated with gender (i.e., being female), the frequency of screen time for homeschooling, the number of life events, and negatively with television viewing. The results of the hierarchical regression analyses (see Table 3) show that the final model with single screen-media activities (Model 3) accounted for 32.6% of the variance in mental health problems at T2. At each step, a significant proportion of the variance was explained. In Model 1, with covariates as the only predictors, being female (β = 0.181, *p* < 0.001) and having experienced one (β = 0.263, *p* < 0.001), two (β = 0.262, *p* < 0.001) or more (β = 0.453, *p* < 0.001) negative life events, and the frequency of using screens for homeschooling (β = 0.119, *p* = 0.002) significantly predicted higher levels of mental health problems at T2. Subjective SES did not show a significant effect, neither did the at-home living situation. In Model 2, overall mental health problems at T1 significantly predicted themselves at T2 (β = 0.371, *p* < 0.001). Significant predictors in Model 1 remained significant in Model 2. In Model 3, including difference indices in screen-media activities, one further significant effect was found: Increased time spent on social media (β = 0.112, *p* = 0.016) was associated with worse mental health at T2. When single screen-media activities were substituted with overall social screen time (i.e., Internet, use, social media use, messging, and smartphone use; Model 4), the effect on mental health problems at T2 was positive but small (β = 0.083, *p* = 0.014), and the model explained 30% of the variance in the outcome.

In further linear regression analyses replicating previous models, we examined the impact of changes in screen-media activities and overall screen time on specific mental health symptoms that significantly worsened from the pre-lockdown to the post-lockdown assessment (see Supplementary Tables 2–7). We thus excluded somatic problems, irritability, and anger, which did not change from T1 to T2. Controlling for covariates, increased time spent on social media significantly and positively predicted depression (β = 0.143, *p* = 0.004) and inattention (β = 0.133, *p* = 0.011) at T2. With these two exceptions, social media use did not predict other mental health symptoms. However, the more time adolescents spent viewing television, the lower were their levels of inattention (β = −0.091, *p* = 0.021) and anxiety (β = −0.093, *p* = 0.014). On the other hand, augmented time spent on video games predicted less depressive symptoms (β = −0.081, *p* = 0.037), sleep problems (β = −0.085, *p* = 0.035), and OCD symptoms (β = −0.096, *p* = 0.024). None of the screen-media activities showed an influence on loneliness. However, when overall social screen time was entered in the analyses, it positively predicted loneliness (β = 0.109, *p* = 0.002), depressive symptoms (β = 0.085, *p* = 0.037), and inattention (β = −0.075, *p* = 0.042). With respect to the covariates, gender (being female) and having experienced two or more life events had a significant effect on all symptoms. However, attention problems were not predicted by gender, and only having experienced more than two life events positively influenced this outcome. Interestingly, the use of screens for homeschooling had a positive and significant effect on specific mental health symptoms, such as anxiety (β = 0.122, *p* = 0.001) and OCD symptoms (β = 0.096, *p* = 0.019). The at-home living situation did not influence any of the outcomes, neither did subjective SES.

## Discussion

In the present study, we investigated the long-term effects of the COVID-19 pandemic in Switzerland on adolescents' mental health by conducting a natural experiment including longitudinal data collected before and after the lockdown. In particular, we estimated how adolescents' mental health changed from Spring 2019 to Autumn 2020, and how overall screen time and specific screen-based activities predicted adolescents' mental health throughout this 1.5-year time period. The present study revealed four major findings.

First, and not surprisingly, adolescents' mental health worsened over time, with a particular increase in internalizing problems such as anxiety and depression, but also inattention, which showed medium to large effect sizes. This result is in line with a systematic review (6) that included 61 articles for a total of 54,999 children and adolescents, showing that anxiety and depressive symptoms were the most reported problems since the COVID-19 outbreak and associated containment measures i.e. lockdowns, closure of schools and workplaces as well as distancing measures, especially in females and adolescents. Peer relationships during adolescent years are of great importance since this developmental period is characterized by a high need of social interaction and interpersonal relationships outside the family to develop personal identity. The interruption of in-person schooling and recreational events likely increased feelings of anxiety and depression and augmented general mental health problems. Additionally, we should consider that the onset of internalizing problems increases as children transition into adolescence (16). Hence, the experience of a stressful life event like the COVID-19 lockdown during Spring 2020 might have increased the likelihood to develop mental health problems, especially in adolescents due to reduced resilience and coping skills, which usually develop during adulthood thanks to the maturation of specific brain regions (56). Also, parental stress and family conflict could have modulated adolescents' stress response and, consequentially, their levels of anxiety and depression during the lockdown (23, 57). Parents' perception of COVID-19 has been found to be related to their children's psychological symptoms, and when family living situation during the lockdown was problematic, adolescents tended to show more emotional problems. Furthermore, inattention increased over time. This result is in line with another study carried out by Orgilés et al. (57), who investigated the emotional impact of quarantine on adolescents living in two of the European countries most affected by COVID-19: Italy and Spain. The authors reported that 76.6% of the sample experienced symptoms such as difficulties in concentrating. This may be mainly due to the disruption of daily routines and the change to homeschooling. However, symptoms of inattention including fatigue and cognitive impairment might also be long-term consequences of infections with the COVID-19 virus (58). However, as we did not assess if they have been infected from COVID-19 during that period, the interpretation of this result is limited.

The second main result is that most screen-media activities increased. Internet and smartphone use showed the highest increment between Spring 2019 and Autumn 2020, with an average leisure time spent on them between 2.5 and 3 hours per day, followed by social media use and messaging. Several studies have indicated increased time spent on screen media during COVID-19 (16, 56). Our result is also in line with another Swiss study that analyzed data from before the pandemic. Using a repeated cross-sectional design, the study showed the highest increment in time spent on online activities in the last 10 years in Switzerland (18). The COVID-19 pandemic accelerated this trend. In the US, early in the COVID-19 pandemic, adolescents' average daily screen time was 7.70 hours per day, whereas pre-pandemic estimates were lower (3.8 hours per day) (59). However, time spent watching television and video gaming decreased in our sample. This result might also be due to the way questions were formulated. For many students, watching TV has been mainly replaced by watching online videos or films or series on streaming platforms such as Netflix and Amazon Prime (which we did not ask). Also, playing video games can be interpreted in many ways, since these days online games also include a social component – for which players are usually continuously connected to each other – but classic video games (as the ones the question refers to) lack that component.

Third, when looking at how differences in screen-media activities impacted mental health symptoms after the COVID-19 lockdown, only social media use showed a positive effect, i.e., adolescents reporting to have increased their time spent on social media use after the onset of the pandemic also showed more frequent symptoms of mental health problems. This result is in line with previous literature indicating small but negative effects of social media use on mental health (e.g., for reviews of reviews) (33, 54, 58–62). Underlying mechanisms are manifold. Social comparison and envy might have been heightened by spending more time on social media (63). Likewise, screen time might have displaced time spent on offline activities such as physical activity and green time that proved to be beneficial for adolescents' mental health (44, 64). Heavy social media use has also been related to a (maladaptive) emotional coping process to escape from negative emotions, especially in response to environmental stressors (65–67). Additionally, the exposure to COVID-19-related news augmented stress by increasing feelings of FoMO (e.g., with respect to COVID-19-related news and updates or about other people's whereabouts) and fostering rumination (41). Despite significant concerns about COVID-19, adolescents might also have other everyday-life worries and issues that did not end with the pandemic but probably increased, thus fueling feelings of anxiety and depression (68). However, greater time spent on watching television and video gaming was related to less mental health problems. One explanation could be that, for example, watching television is an activity that adolescents might carry out together with other family members in a specific space, e.g., in the living room, and time determined by broadcasters' TV programming schedule. Hence, it is more routinized, and it might give them a structured time of consumption of media contents. Routined activities were found to be beneficial for health outcomes in a context where any kind of structure has been lost (69, 70). Indeed, according to the *structured days hypothesis*, a “pre-planned, segmented, and adult-supervised compulsory environment plays an overall protective role” (p. 2, 69) against the occurrence of negative health outcomes (including obesogenic activities like long time spent in front of screens). Also, according to a review and meta-analysis (69), screen time is less healthy on less structured days. In addition, the positive aspect of video gaming could be mainly related to entertainment reasons but also to the possibility to completely detach from COVID-19-related news and worries and find momentary relief in the gaming space. In fact, although escaping from negative emotions is a criterion used to diagnose Online gaming disorder, it can also be interpreted as a problem-oriented coping mechanism, which aims to change stressful situations, or as an avoidance strategy, which aims to orient away from the stressful situation (71). In this regard, video gaming could have facilitated coping strategies, which already exist without screen media, or it could stand as a new coping strategy itself, thus belonging to a different dimension that should be further conceptualized – especially in the context of life events like a pandemic. That is particularly relevant for the adolescent period, during which young people are still developing their coping styles (72).

Last, among covariates, being female was a consistent risk factor for developing mental health problems. From a biological viewpoint, girls at that age are more at risk of internalizing problems like depression (73, 74) as they tend to produce an increased cortisol response to stressful events (75), and pubertal hormone changes, e.g., estradiol, are also associated with mental health problems in girls (76). Girls carrying the short 5-HTTLPR allele (77), a genetic variant associated with various mood and anxiety problems (78), react differently with respect to males (79) to environmental (social) stress factors, thus they are at higher risk for depressive symptoms. From the socio-cultural viewpoint, female adolescents show higher interpersonal dependence, more self-image and self-esteem concerns, and a higher need for external approval and success (80). Also, the experience of two or more life events – in addition to the COVID-19 lockdown in Spring 2020 – was related to worse mental health outcomes in Autumn 2020. This should be considered in the context of the adolescent development period, during which the hypothalamic-pituitary-adrenal axis (responsible for the biological response to stress through the release of cortisol) is still maturing and adapting to the current environment, and stress response is repeatedly heightened with respect to adulthood (81). We should recall that almost 32% of adolescents in our sample reported the worsening of a significant relationship since the onset of the pandemic. Although we do not know which relationship in particular worsened, we know from other studies that the lockdown increased parent-child conflict as family members were confined to oftentimes small spaces and had less room for privacy, which is particularly important during adolescence (82, 83). In addition, it is likely that heightened stress experienced by parents due to the COVID-19 pandemic negatively impacted the parent-adolescent relationship (84). Furthermore, intimate friendships and romantic relationships were put to the test due to strict social (physical) distancing measures during the early lockdown in Switzerland. Eventually, the use of digital devices, especially laptops and smartphones, for homeschooling revealed a negative influence on two specific mental health problems: anxiety and OCD symptoms. School is a crucial component in sustaining adolescents' physical, emotional, social, and moral development, however, according to our results, the use of digital devices in this context contributed to the spread of anxiety and worry, and this may be due to the fact that neither schools nor students were prepared for online or hybrid forms of schooling. This lack of preparedness likely increased uncertainty and anxiety, especially in the first year high school characterized by new school structures, classmates, and teachers, during which our sample had to face the early phases of the COVID-19 pandemic.

## Limitations and future directions

Some methodological limitations should be acknowledged. Given the topical variety of the larger longitudinal study from which the data were taken, we did not include any measure referring to the COVID-19 pandemic, e.g., COVID-19-related stress or infection with the virus. Additionally, some screen-media activities, like watching television or video gaming, were formulated in a way that no longer captures the actual consumption of mostly online media content. For example, we did not consider other related activities like streaming or staying socially connected while using online video games. In addition, we did not measure specific activities and processes such as social comparison or FoMO as potential mechanisms between significant relationships. Hence, further (longitudinal) studies should implement more information about the context and a more detailed screen-media assessment (85). Similarly, we did not assess the subjective SES in a comprehensive way. According to the social determinat approach to health outcomes (86), more information on the socio-economic status, community, and societal characteristics should be collected. In addition, our sample might not be representative of the more vulnerable and underprivileged populations (e.g. under-represented races and ethnicities, lesbian, gay, bisexual, transgender & queer (LGBTQ) communities), thus contributing to what has been called “data absenteeism” during the COVID-19 pandemic, defined as “absence or limits of data on groups experiencing social vulnerability” [p. 208, (87)]. Hence, we suggest that future studies focus on the “hardly reached” populations. At the same time, although we included a comprehensive assessment of mental health, some symptoms that the pandemic might exacerbate were not investigated, for example, the presence of eating disorders, suicidal ideation, psychotic symptoms, post-traumatic stress disorder, and mania. Furthermore, the present cohort of students does not extend to other age groups, which showed differential effects of social media use (88). Although the dropout of participants was mainly due to the change of school (from middle to high school), it is possible that some students dropped out because they were particularly suffering the consequences of the pandemic in the post-lockdown assessment. Finally, we did not consider if there were any students with special educational needs and disabilities and/or neurodevelopmental disorders that might have experienced more psychological stress since they prefer routine and predictable environments (6). Challenges of remote learning are especially difficult for disadvantaged and minority families and likely augmented social and economic disparities since young people remain left behind in their education (89). Hence, future studies should better consider specific disabilities in studying the consequences of screen media on mental health during COVID-19.

## Conclusion

Mental wellbeing in adolescents significantly decreased from the pre-pandemic to the post-lockdown period in Switzerland. At the same time, screen-media activities with a social component, such as smartphone use, social media use, messaging, and Internet use, increased. The increase in social media use was significantly associated with worse mental health in the post-lockdown period, even after controlling for other significant predictors, including mental health before the onset of the COVID-19 pandemic, being female, the presence of at least one life event on top of the pandemic, the at-home living situation, and the use of screen media for homeschooling. These findings shed light on the impact of the early phases of the COVID-19 pandemic on adolescents, who are in a crucial developmental period and particularly vulnerable, thus requiring more attention both among researchers and policymakers.

## Data availability statement

The original contributions presented in the study are included in the article/Supplementary materials, further inquiries can be directed to the corresponding author.

## Ethics statement

Ethical review and approval were not required for the study on human participants in accordance with the local legislation and institutional requirements. However, the regional education administration of Canton Ticino and the Ethics Committee of the USI Università della Svizzera italiana (Switzerland) approved this study design. Written informed consent from participants' legal guardian/next of kin was not required to participate in this study in accordance with the national legislation and the institutional requirements. However, consent was implied *via* completion of the questionnaire.

## Author contributions

LM and A-LC conceived the project, collected the data, and provided funding. LM performed the statistical analysis and wrote the first draft of the manuscript. A-LC, RM, and KV wrote sections of the manuscript. All authors contributed to manuscript revision, read, and approved the submitted version.

## Funding

This study was funded by the Swiss National Science Foundation (Grant Nos. 10001C_175874 and P500PS_202974).

## Conflict of interest

The authors declare that the research was conducted in the absence of any commercial or financial relationships that could be construed as a potential conflict of interest.

## Publisher's note

All claims expressed in this article are solely those of the authors and do not necessarily represent those of their affiliated organizations, or those of the publisher, the editors and the reviewers. Any product that may be evaluated in this article, or claim that may be made by its manufacturer, is not guaranteed or endorsed by the publisher.
