# Supplementary material for: Screen time and adolescents' mental health before and after the COVID-19 lockdown in Switzerland: A natural experiment
Source: Front Psychiatry. 2022 Nov 16;13:981881. doi: 10.3389/fpsyt.2022.981881 (PMC9709147; doi:10.3389/fpsyt.2022.981881)
Supplement: Supplementary file 1 [file Data_Sheet_1.docx]

***Supplementary Material***

**Table 1.** List of items in the questionnaire and response options.

| **Problemi psicologici** |
| --- |
| ***Mental health problems*** |
| Durante l’ultimo mese, quanto spesso ti sono capitate le seguenti cose? |
| *During the past month, how much (or how often) have you experienced any of these symptoms?* |
|  |
| (1) Mai (2) Raramente (meno di un giorno o due) (3) Per alcuni giorni (4) Per più della metà dei giorni (5) Quasi ogni giorno |
| *(1)Never (2) Rarely (less than a day or two) (3) For a few days (4) For more than half the days (5) Almost every day* |
|  |
| Dolori somatici |
| *Somatic symptoms* |
| 1. Ho avuto dolori allo stomaco, alla testa o altri dolori o malesseri. |
| *I have been bothered by stomachaches, headaches, or other aches and pains.* |
|  |
| Inattenzione |
| *Inattention* |
| 1. Ho avuto difficoltà a organizzare compiti e attività. |
| *I had a hard time organizing tasks and activities.* |
| 1. Mi sono distratto/a facilmente. |
| *I have been distracted easily.* |
| 1. Ho avuto difficoltà a prestare attenzione mentre ero in classe, facevo i compiti, leggevo o giocavo.*I have been bothered by not being able to pay attention when I was in class or doing homework or reading a book or playing a game.* |
|  |
| Irritabilità |
| *Irritability* |
| 1. Ero più irritato/a o facilmente irritabile rispetto al solito. |
| *I felt more irritated or easily annoyed than usual* |
|  |
| Rabbia |
| *Anger* |
| 1. Ero arrabbiato/a, ho perso il controllo. |
| *I felt angry or lost my temper.* |
|  |
| Problemi di sonno |
| *Sleep Problems* |
| 1. Ho avuto difficoltà a dormire tutta la notte senza svegliarmi. |
| *I had a hard time sleeping through the night without waking up.* |
| 1. Ho avuto difficoltà ad addormentarmi. |
| *I had a hard time falling asleep.* |
| 1. Ho avuto problemi a svegliarmi presto. |
| *I was having trouble waking up early.* |
|  |
| Ansia |
| *Anxiety* |
| 1. Mi sono sentito/a in ansia, irrequieto/a, agitato/a. |
| *I felt nervous, anxious, or scared.* |
| 1. Non riuscivo a smettere di preoccuparmi per quello che dovevo fare (es. per la scuola). |
| *I was not able to stop worrying for the things I had to do (es. for school)* |
|  |
| Sintomi ossessivi-compulsivi |
| *Obsessive-compulsive disorder symptoms* |
| 1. Ho sentito il bisogno di fare certe cose per più volte (es. lavarmi le mani, controllare, ordinare). |
| *I felt I had to do things in a certain way (es. washing hands, counting, ordering).* |
|  |
| **Solitudine** |
| *Loneliness* |
| In generale, quanto spesso … |
| *Usually, how often...* |
|  |
| 1. Praticamente mai (2) Raramente (3) A volte (4) Spesso |
| *(1) Hardly ever (2) Rarely (3) Sometimes (4) Often* |
|  |
| 1. Ti mi manca la compagnia di qualcuno? |
| *Do you feel that you lack companionship?* |
|  |
| 1. Ti senti escluso/a? |
| *Do you feel left out?* |
|  |
| 1. Ti senti isolato/a dagli altri (pur essendo in compagnia)? |
| *Do you feel isolated from others (while being in company)?* |
|  |
| **Depressione** |
| ***Depression*** |
|  |
| Se pensi alla settimana appena trascorsa, quanto hai provato i seguenti sentimenti? |
| *If you think about the past week, how often did you experience the following feelings?* |
|  |
| 1. Per niente (2) Un po’ (3) Abbastanza (4) Tanto |
| *(1) Not at all (2) Sometimes (3) Often (4) Always* |
|  |
| 1. Non riuscivo ad essere felice, anche quando la mia famiglia e i miei amici hanno cercato di aiutarmi a sentirmi meglio. |
| *I couldn't be happy, even when my family and friends tried to help me feel better.* |
|  |
| 1. Mi sono sentito/a giù e non contento/a. |
| *I felt sad and unhappy.* |
|  |
| 1. Non riuscivo più a fare bene le cose. |
| *I couldn't do things right anymore.* |
|  |
| 1. Ero angosciato/a, preoccupato/a. |
| *I was distressed, worried.* |
|  |
| 1. Mi sono sentito/a solo/a, come se non avessi amici. |
| *I felt lonely, like I didn't have any friends.* |
|  |
| 1. Mi veniva da piangere. |
| *I felt like crying.* |
|  |
| 1. Mi sono sentito/a triste. |
| *I felt depressed.* |
|  |
| **Uso dei media digitali** |
| ***Screen media use*** |
|  |
| Risposte |
| *Answer options* |
| 1. Mai (1) Fino a mezz’ ora (2) Da ½ ora a 1 ora |
| (3)Da 1 a 1½ ore (4) Da 1½ a 2 ore (5) Da 2 a 3 ore |
| (6) Da 3 a 4 ore (7)Da 4 a 5 ore (8)Più di 5 ore |
|  |
| *0) Never (1) Up to half an hour ( 2) From ½ hour to 1 hour* |
| *(3) From 1 to 1½ hours (4) From 1½ to 2 hours (5) From 2 to 3 hours* |
| *(6) From 3 to 4 hours (7) From 4 to 5 hours (8) More than 5 hours* |
|  |
| Uso di Internet |
| *Internet use* |
| 1. Quanto tempo usi internet (in generale) durante un normale giorno di scuola/lavoro e durante un giorno del weekend (es. sabato)? (Pensa al computer fisso e portatile, al tablet e allo smartphone, inclusi quelli dei tuoi genitori, degli amici o di altri) |
| *How long do you use the internet (in general) on a normal school/work day and on a weekend day (e.g. Saturday)? (eg, desktop and laptop computers, tablets and smartphones, including those of your parents, friends or others)* |
|  |
| Smartphone use |
| *Smartphone use* |
| 1. Pensando solo allo smartphone, quanto tempo lo usi durante un normale giorno di scuola/lavoro e durante un giorno del weekend (es. sabato)? |
| *Thinking only about the smartphone, how much time do you use it during a normal school/work day and during a weekend day (eg Saturday)?* |
|  |
| Social media |
| *Social media* |
| 1. Per quanto tempo usi i social network (es. Instagram, Snapchat – No WhatsApp) durante un normale giorno di scuola/lavoro e durante un giorno del weekend (es. sabato)? |
| *How long do you use social networks (eg Instagram, Snapchat - No WhatsApp) during a normal school / work day and during a weekend day (eg Saturday)?* |
|  |
| Messaggistica |
| *Messaging* |
| 1. Per quanto tempo usi la messaggistica istantanea (es. WhatsApp, Telegram) durante un normale giorno di scuola/ lavoro e durante un giorno del weekend (es. sabato)? |
| *How long do you use instant messaging (eg WhatsApp, Telegram) during a normal school / work day and during a weekend day (eg Saturday)?* |
|  |
| Videogiochi |
| *Video games* |
| 1. Quanto tempo usi i videogiochi (tramite console, PC, smartphone, tablet e online in generale) durante un normale giorno di scuola/lavoro e durante un giorno del weekend (es. sabato)? |
| *How long do you use video games (via console, PC, smartphone, tablet and online in general) during a normal school / work day and during a weekend day (eg Saturday)?* |
|  |
| Televisione |
| *Television viewing* |
| 1. Per quanto tempo guardi la televisione durante un normale giorno di scuola/lavoro e durante un giorno del weekend (es. sabato)? |
| *How long do you watch television on a normal school / work day and on a weekend day (e.g. Saturday)?* |

Table 2. Screen-media activities and **depressive symptoms.**

|  | **Model 1** | | | | **Model 2** | | | | **Model 3** | | | | | | **Model 4** | | | |  |
| --- | --- | --- | --- | --- | --- | --- | --- | --- | --- | --- | --- | --- | --- | --- | --- | --- | --- | --- | --- |
|  | B | (SE) | Beta | p-value | B | (SE) | Beta | p-value | | B | SE | Beta | p-value | B | | (SE) | Beta | p-value | |
| (Constant) | .355 | .152 |  | .020 | .205 | .148 |  | .168 | | .157 | .148 |  | .290 | .220 | | .139 |  | .113 | |
| Gender (1=female) | .347 | .066 | .209 | **.000** | .289 | .064 | .174 | **.000** | | .312 | .066 | .187 | **.000** | .268 | | .060 | .161 | **.000** | |
| Life-changing events at (n=1)_T2_ | .309 | .081 | .162 | **.000** | .279 | .078 | .147 | **.000** | | .308 | .078 | .162 | **.000** | .303 | | .072 | .161 | **.000** | |
| Life -changing events (n≥2)_T2_ | .535 | .079 | .294 | **.000** | .443 | .078 | .244 | **.000** | | .458 | .077 | .252 | **.000** | .455 | | .073 | .250 | **.000** | |
| Subjective SES at T2 (1=not wealthy)_T2_ | .017 | .068 | .010 | .800 | .035 | .066 | .021 | .592 | | .029 | .066 | .017 | .663 | -.030 | | .061 | -.018 | .615 | |
| At-home living situation (1= with 3 or more other people)_T2_ | .012 | .070 | .007 | .867 | .057 | .068 | .032 | .403 | | .059 | .067 | .033 | .384 | .043 | | .064 | .024 | .506 | |
| Use of screens for home schooling_T2_ | .096 | .045 | .085 | .**031** | .086 | .043 | .075 | .**047** | | .077 | .043 | .067 | .077 | .087 | | .040 | .077 | .**031** | |
| Depressive symptoms _T1_ |  |  |  |  | .299 | .047 | .252 | .000 | | .319 | .047 | .269 | **.000** | .284 | | .043 | .246 | .**000** | |
| Internet use_ΔT2-T1_ |  |  |  |  |  |  |  |  | | -.006 | .021 | -.015 | .767 |  | |  |  |  | |
| Smartphone use_ΔT2-T1_ |  |  |  |  |  |  |  |  | | -.014 | .023 | -.033 | .546 |  | |  |  |  | |
| Social media use_ΔT2-T1_ |  |  |  |  |  |  |  |  | | .063 | .022 | .143 | **.004** |  | |  |  |  | |
| Messaging_ΔT2-T1_ |  |  |  |  |  |  |  |  | | .012 | .021 | .028 | .564 |  | |  |  |  | |
| Video gaming_ΔT2-T1_ |  |  |  |  |  |  |  |  | | -.032 | .016 | -.081 | **.037** |  | |  |  |  | |
| Television viewing_ΔT2-T1_ |  |  |  |  |  |  |  |  | | -.031 | .020 | -.062 | .117 |  | |  |  |  | |
| Overall social screen time_ΔT2-T1_ |  |  |  |  |  |  |  |  | |  |  |  |  | .039 | | .019 | .075 | **.037** | |
| R squared | .143** | | | | .201** | | | | .217** | | | | | | .194** | | | |  |

Legend: ** p<.01

Table 3. Screen-media activities and **loneliness**

|  | Model 1 | | | | Model 2 | | | | Model 3 | | | | Model 4 | | | |
| --- | --- | --- | --- | --- | --- | --- | --- | --- | --- | --- | --- | --- | --- | --- | --- | --- |
|  | B | (SE) | Beta | p-value | B | (SE) | Beta | p-value | B | SE | Beta | p-value | B | (SE) | Beta | p-value |
| (Constant) | 1.914 | .143 |  | .000 | 1.329 | .148 |  | .000 | 1.299 | .149 |  | .000 | 1.244 | .141 |  | .000 |
| Gender (1=female) | .291 | .062 | .191 | **.000** | .170 | .060 | .112 | **.005** | .176 | .061 | .116 | **.004** | .137 | .056 | .088 | **.015** |
| Life-changing events at (n=1)_T2_ | .201 | .076 | .115 | **.009** | .135 | .072 | .078 | .059 | .144 | .072 | .083 | .045 | .142 | .066 | .082 | **.032** |
| Life -changing events (n≥2)_T2_ | .348 | .075 | .209 | **.000** | .278 | .070 | .167 | **.000** | .281 | .070 | .168 | .**000** | .310 | .066 | .183 | **.000** |
| Subjective SES at T2 (1=not wealthy)_T2_ | .036 | .064 | .023 | .569 | .031 | .060 | .020 | .607 | .036 | .060 | .023 | .553 | -.018 | .055 | -.012 | .742 |
| At-home living situation (1= with 3 or more other people)_T2_ | .048 | .066 | .030 | .463 | .056 | .062 | .035 | .362 | .049 | .062 | .030 | .430 | .059 | .059 | .036 | .314 |
| Use of screens for home schooling_T2_ | .036 | .042 | .034 | .397 | .014 | .039 | .014 | .713 | .008 | .040 | .008 | .833 | .024 | .037 | .023 | .522 |
| Loneliness _T1_ |  |  |  |  | .341 | .038 | .356 | **.000** | .349 | .038 | .365 | .**000** | .362 | .035 | .374 | **.000** |
| Internet use_ΔT2-T1_ |  |  |  |  |  |  |  |  | -.018 | .019 | -.046 | .360 |  |  |  |  |
| Smartphone use_ΔT2-T1_ |  |  |  |  |  |  |  |  | .035 | .021 | .089 | .098 |  |  |  |  |
| Social media use_ΔT2-T1_ |  |  |  |  |  |  |  |  | .025 | .020 | .062 | .221 |  |  |  |  |
| Messaging_ΔT2-T1_ |  |  |  |  |  |  |  |  | .006 | .020 | .016 | .749 |  |  |  |  |
| Video gaming_ΔT2-T1_ |  |  |  |  |  |  |  |  | .005 | .018 | .011 | .784 |  |  |  |  |
| Television viewing_ΔT2-T1_ |  |  |  |  |  |  |  |  | -.009 | .014 | -.024 | .530 |  |  |  |  |
| Overall social screen time_ΔT2-T1_ |  |  |  |  |  |  |  |  |  |  |  |  | .053 | .017 | .109 | **.002** |
| R squared | .082 | | | | .198 | | | | .204 | | | | .211 | | | |

Legend: ** p<.01

Table 4. Screen-media activities and **inattention.**

|  | Model 1 | | | | Model 2 | | | | Model 3 | | | | Model 4 | | | |
| --- | --- | --- | --- | --- | --- | --- | --- | --- | --- | --- | --- | --- | --- | --- | --- | --- |
|  | B | (SE) | Beta | p-value | B | (SE) | Beta | p-value | B | SE | Beta | p-value | B | (SE) | Beta | p-value |
| (Constant) | 1.876 | .177 |  | .000 | 1.176 | .186 |  | .000 | 1.084 | .186 |  | .000 | 1.246 | .175 |  | .000 |
| Gender (1=female) | .143 | .077 | .077 | .065 | .085 | .073 | .046 | .247 | .098 | .075 | .053 | .189 | .107 | .069 | .057 | .121 |
| Life-changing events at (n=1)_T2_ | .213 | .094 | .100 | **.025** | .137 | .089 | .065 | .126 | .147 | .089 | .069 | .101 | .162 | .083 | .077 | .052 |
| Life -changing events (n≥2)_T2_ | .443 | .093 | .218 | **.000** | .337 | .088 | .166 | **.000** | .339 | .088 | .167 | **.000** | .327 | .083 | .159 | **.000** |
| Subjective SES at T2 (1=not wealthy)_T2_ | .130 | .079 | .068 | .101 | .091 | .075 | .048 | .222 | .083 | .075 | .044 | .265 | .072 | .070 | .038 | .302 |
| At-home living situation (1= with 3 or more other people)_T2_ | .100 | .082 | .051 | .221 | .100 | .077 | .051 | .195 | .100 | .076 | .051 | .191 | .037 | .074 | .018 | .615 |
| Use of screens for home schooling_T2_ | .100 | .052 | .079 | .056 | .098 | .049 | .077 | .**046** | .088 | .049 | .070 | .074 | .067 | .046 | .053 | .146 |
| Inattention _T1_ |  |  |  |  | .360 | .043 | .332 | **.000** | .381 | .043 | .352 | .**000** | .372 | .041 | .341 | **.000** |
| Internet use_ΔT2-T1_ |  |  |  |  |  |  |  |  | .038 | .024 | .083 | .111 |  |  |  |  |
| Smartphone use_ΔT2-T1_ |  |  |  |  |  |  |  |  | -.009 | .027 | -.019 | .732 |  |  |  |  |
| Social media use_ΔT2-T1_ |  |  |  |  |  |  |  |  | .065 | .025 | .133 | **.011** |  |  |  |  |
| Messaging_ΔT2-T1_ |  |  |  |  |  |  |  |  | -.035 | .024 | -.071 | .149 |  |  |  |  |
| Video gaming_ΔT2-T1_ |  |  |  |  |  |  |  |  | -.002 | .022 | -.004 | .928 |  |  |  |  |
| Television viewing_ΔT2-T1_ |  |  |  |  |  |  |  |  | -.041 | .018 | -.091 | **.021** |  |  |  |  |
| Overall social screen time_ΔT2-T1_ |  |  |  |  |  |  |  |  |  |  |  |  | .044 | .022 | .075 | **.042** |
| R squared | .058 | | | | .163 | | | | .180 | | | | .160 | | | |

Legend: ** p<.01

Table 5. Screen-media activities and **sleep problems**.

| \ | Model 1 | | | | Model 2 | | | | Model 3 | | | | Model 4 | | | |
| --- | --- | --- | --- | --- | --- | --- | --- | --- | --- | --- | --- | --- | --- | --- | --- | --- |
|  | B | (SE) | Beta | p-value | B | (SE) | Beta | p-value | B | SE | Beta | p-value | B | (SE) | Beta | p-value |
| (Constant) | 1.808 | .175 |  | .000 | 1.184 | .181 |  | .000 | 1.120 | .183 |  | .000 | 1.243 | .170 |  | .000 |
| Gender (1=female) | .403 | .077 | .215 | **.000** | .304 | .073 | .162 | **.000** | .339 | .075 | .180 | **.000** | .305 | .069 | .160 | **.000** |
| Life-changing events at (n=1)_T2_ | .256 | .094 | .119 | **.006** | .221 | .088 | .103 | **.013** | .247 | .089 | .115 | **.006** | .302 | .082 | .141 | **.000** |
| Life -changing events (n≥2)_T2_ | .431 | .092 | .210 | **.000** | .344 | .087 | .168 | **.000** | .360 | .087 | .175 | **.000** | .310 | .082 | .148 | **.000** |
| Subjective SES at T2 (1=not wealthy)_T2_ | -.002 | .078 | -.001 | .977 | -.023 | .074 | -.012 | .753 | -.024 | .075 | -.012 | .752 | -.039 | .069 | -.020 | .570 |
| At-home living situation (1= with 3 or more other people)_T2_ | .109 | .081 | .055 | .177 | .079 | .076 | .040 | .304 | .086 | .076 | .043 | .263 | .030 | .073 | .015 | .680 |
| Use of screens for home schooling_T2_ | .068 | .052 | .053 | .187 | .077 | .049 | .060 | .115 | .077 | .049 | .060 | .119 | .056 | .046 | .044 | .217 |
| Sleep problems _T1_ |  |  |  |  | .311 | .037 | .325 | **.000** | .312 | .038 | .325 | **.000** | .328 | .035 | .341 | **.000** |
| Internet use_ΔT2-T1_ |  |  |  |  |  |  |  |  | -.022 | .024 | -.047 | .356 |  |  |  |  |
| Smartphone use_ΔT2-T1_ |  |  |  |  |  |  |  |  | .033 | .027 | .068 | .210 |  |  |  |  |
| Social media use_ΔT2-T1_ |  |  |  |  |  |  |  |  | .027 | .025 | .055 | .280 |  |  |  |  |
| Messaging_ΔT2-T1_ |  |  |  |  |  |  |  |  | -.015 | .024 | -.031 | .529 |  |  |  |  |
| Video gaming_ΔT2-T1_ |  |  |  |  |  |  |  |  | -.047 | .022 | -.085 | **.035** |  |  |  |  |
| Television viewing_ΔT2-T1_ |  |  |  |  |  |  |  |  | -.012 | .018 | -.026 | .505 |  |  |  |  |
| Overall social screen time_ΔT2-T1_ |  |  |  |  |  |  |  |  |  |  |  |  | .010 | .021 | .017 | .630 |
| R squared | .095** | | | | .194** | | | | .198** | | | | .199** | | | |

Legend: ** p<.01

Table 6. Screen-media activities and **anxiety**.

|  | Model 1 | | | | Model 2 | | | | Model 3 | | | | Model 4 | | | |
| --- | --- | --- | --- | --- | --- | --- | --- | --- | --- | --- | --- | --- | --- | --- | --- | --- |
|  | B | (SE) | Beta | p-value | B | (SE) | Beta | p-value | B | SE | Beta | p-value | B | (SE) | Beta | p-value |
| (Constant) | 1.580 | .217 |  | .000 | .925 | .219 |  | .000 | .902 | .220 |  | .000 | .950 | .206 |  | .000 |
| Gender (1=female) | .674 | .095 | .280 | **.000** | .485 | .092 | .202 | **.000** | .480 | .094 | .200 | **.000** | .419 | .086 | .175 | **.000** |
| Life-changing events at (n=1)_T2_ | .231 | .116 | .084 | **.046** | .201 | .109 | .073 | **.066** | .219 | .110 | .080 | **.047** | .242 | .101 | .090 | **.017** |
| Life -changing events (n≥2)_T2_ | .580 | .114 | .221 | **.000** | .469 | .108 | .178 | **.000** | .484 | .108 | .184 | **.000** | .459 | .101 | .175 | **.000** |
| Subjective SES at T2 (1=not wealthy)_T2_ | .040 | .097 | .016 | .677 | .039 | .091 | .016 | .671 | .018 | .092 | .007 | .847 | -.035 | .085 | -.014 | .680 |
| At-home living situation (1= with 3 or more other people)_T2_ | .124 | .100 | .049 | .217 | .151 | .094 | .059 | .111 | .151 | .094 | .059 | .111 | .140 | .089 | .055 | .118 |
| Use of screens for home schooling_T2_ | .223 | .064 | .136 | **.001** | .202 | .060 | .123 | .**001** | .200 | .061 | .122 | **.001** | .201 | .056 | .125 | **.000** |
| Anxiety _T1_ |  |  |  |  | .364 | .043 | .326 | **.000** | .361 | .043 | .323 | **.000** | .372 | .040 | .335 | **.000** |
| Internet use_ΔT2-T1_ |  |  |  |  |  |  |  |  | -.009 | .029 | -.015 | .754 |  |  |  |  |
| Smartphone use_ΔT2-T1_ |  |  |  |  |  |  |  |  | -.025 | .033 | -.040 | .447 |  |  |  |  |
| Social media use_ΔT2-T1_ |  |  |  |  |  |  |  |  | .035 | .031 | .055 | .264 |  |  |  |  |
| Messaging_ΔT2-T1_ |  |  |  |  |  |  |  |  | .030 | .030 | .047 | .321 |  |  |  |  |
| Video gaming_ΔT2-T1_ |  |  |  |  |  |  |  |  | -.004 | .028 | -.006 | .875 |  |  |  |  |
| Television viewing_ΔT2-T1_ |  |  |  |  |  |  |  |  | -.054 | .022 | -.093 | **.014** |  |  |  |  |
| Overall social screen time_ΔT2-T1_ |  |  |  |  |  |  |  |  |  |  |  |  | .011 | .026 | .014 | .682 |
| R squared | .153** | | | | .250** | | | | .254** | | | | .236** | | | |

Legend: ** p<.01

Table 7. Screen-media activities and **OCD symptoms.**

|  | Model 1 | | | | Model 2 | | | | Model 3 | | | | Model 4 | | | |
| --- | --- | --- | --- | --- | --- | --- | --- | --- | --- | --- | --- | --- | --- | --- | --- | --- |
|  | B | (SE) | Beta | p-value | B | (SE) | Beta | p-value | B | SE | Beta | p-value | B | (SE) | Beta | p-value |
| (Constant) | 1.500 | .239 |  | **.000** | 1.012 | .252 |  | .000 | .939 | .252 |  | .000 | 1.110 | .230 |  | .000 |
| Gender (1=female) | .385 | .105 | .154 | **.000** | .343 | .103 | .137 | .001 | .413 | .105 | .166 | **.000** | .323 | .095 | .132 | **.001** |
| Life-changing events at (n=1)_T2_ | .316 | .129 | .111 | **.014** | .266 | .126 | .093 | .035 | .312 | .126 | .109 | **.014** | .253 | .114 | .091 | **.027** |
| Life -changing events (n≥2)_T2_ | .303 | .126 | .111 | **.016** | .250 | .123 | .091 | .043 | .272 | .123 | .100 | **.027** | .182 | .113 | .068 | .109 |
| Subjective SES at T2 (1=not wealthy)_T2_ | -.071 | .108 | -.028 | .507 | -.046 | .105 | -.018 | .663 | -.025 | .106 | -.010 | .811 | -.110 | .096 | -.044 | .251 |
| At-home living situation (1= with 3 or more other people)_T2_ | .084 | .111 | .032 | .451 | .093 | .108 | .035 | .389 | .089 | .108 | .034 | .410 | .019 | .101 | .007 | .854 |
| Use of screens for home schooling_T2_ | .170 | .070 | .100 | **.016** | .181 | .069 | .107 | **.009** | .163 | .069 | .096 | **.019** | .179 | .063 | .108 | **.005** |
| OCD symptoms _T1_ |  |  |  |  | .233 | .044 | .214 | **.000** | .242 | .044 | .223 | **.000** | .216 | .041 | .203 | **.000** |
| Internet use_ΔT2-T1_ |  |  |  |  |  |  |  |  | -.031 | .033 | -.051 | .349 |  |  |  |  |
| Smartphone use_ΔT2-T1_ |  |  |  |  |  |  |  |  | .046 | .037 | .071 | .216 |  |  |  |  |
| Social media use_ΔT2-T1_ |  |  |  |  |  |  |  |  | .059 | .035 | .090 | .095 |  |  |  |  |
| Messaging_ΔT2-T1_ |  |  |  |  |  |  |  |  | .026 | .034 | .040 | .437 |  |  |  |  |
| Video gaming_ΔT2-T1_ |  |  |  |  |  |  |  |  | -.071 | .031 | -.096 | **.024** |  |  |  |  |
| Television viewing_ΔT2-T1_ |  |  |  |  |  |  |  |  | -.005 | .025 | -.008 | .842 |  |  |  |  |
| Overall social screen time_ΔT2-T1_ |  |  |  |  |  |  |  |  |  |  |  |  | .046 | .030 | .060 | .119 |
| R squared | .048 | | | | .092 | | | | .106 | | | |  | | | |

Legend: ** p<.01
